# Living Well and Long

**Published:** 1873-02

**Authors:** 


### LIVING WELL AND LONG.

Some men have such a gift for making money, that they are ready to retire from business by the time they are forty; multitudes could do it to-day, and have a clear income from investments in United States securities of two thousand dollars a year. Suppose such men were to qualify themselves for "lay preachers," or Methodist circuit riders, or itinerant missionaries, and thus fulfill the Master's command literally,

"AS YE GO, PREACH,"

thus gloriously giving their time and talents to God and humanity at their own charges, what a grand ending would such a life be in physical health and moral power? No stigma from the low and ignorant of

PREACHING FOR MONEY; theirs would be a disinterestedness like unto that of the "Son of man." This traveling day by day in the open air would cure dyspepsia, would improve the appetite, would energize the digestion, would purify the blood, would give stability to the constitution; the mind would not be less benefited; there would be opportunity for study and reflection on horseback, and for that turning the eyes within so promotive of heavenly contemplation; while, mingling with a great variety of men and women, an insight would be given into human nature and human character, which would give an efficiency in piercing the heart between the

JOINTS OF THE HARNESS, which no other training can give. It made Patrick Henry the unrivaled orator that he was, and everywhere and in all ages they have been the greatest masters of men who have most mingled with men.

Suppose a man gave ten years to the work of the ministry, his whole heart and soul enthusiastically engaged in it, what a "summing up" there would be, at the day of judgment of souls saved by such instrumentalities as

STARS IN THE CROWN

of their rejoicings before an assembled world of men and angels!

The Rev. John Jacob Hood, of Synnfield, Center Massachusetts, is now eighty-one years old; he rides three miles to his own church every Sunday, where he preaches two sermons, and instructs a Sunday-school class. He writes one of the sermons every week, and a good, whole-souled, warm-hearted sermon it is too. He began to preach at the age of sixty-eight years, when most old men give up to die. Loafers die early; they live longest who are busiest, and they are the most happy who are the most active.

